# Dysbiosis of Gut Microbiota Associated with Clinical Parameters in Polycystic Ovary Syndrome

**DOI:** 10.3389/fmicb.2017.00324

**Published:** 2017-02-28

**Authors:** Rui Liu, Chenhong Zhang, Yu Shi, Feng Zhang, Linxia Li, Xuejiao Wang, Yunxia Ling, Huaqing Fu, Weiping Dong, Jian Shen, Andrew Reeves, Andrew S. Greenberg, Liping Zhao, Yongde Peng, Xiaoying Ding

**Affiliations:** ^1^State Key Laboratory of Microbial Metabolism, School of Life Sciences and Biotechnology, Shanghai Jiao Tong UniversityShanghai, China; ^2^Department of Endocrinology and Metabolism, Qidong People’s HospitalJiangsu, China; ^3^Department of Obstetrics and Gynecology, Shanghai General Hospital, Shanghai Jiao Tong University School of MedicineShanghai, China; ^4^Department of Endocrinology and Metabolism, Shanghai General Hospital, Shanghai Jiao Tong University School of MedicineShanghai, China; ^5^Obesity and Metabolism Laboratory, Jean-Mayer USDA Human Nutrition Research Center on Aging at Tufts University, BostonMA, USA

**Keywords:** gut microbiota, polycystic ovary syndrome, obesity, testosterone, ghrelin, serotonin

## Abstract

Polycystic ovary syndrome (PCOS) is a common endocrine and metabolic disorder in women. Gut microbiota has been implicated to play a critical role in metabolic diseases and may modulate the secretion of mediators of the brain–gut axis. Interaction between gut microbiota and the endocrine and biochemical disturbances in PCOS still remains elusive. Here, we showed an altered gut microbiota significantly correlated with PCOS phenotype. There were 33 patients with PCOS (non-obese PCOS individuals, PN, *n* = 12; obese PCOS individuals, PO, *n* = 21) as well as 15 control subjects (non-obese control individuals, CN, *n* = 9; obese control individuals, CO, *n* = 6) enrolled in our study. The plasma levels of serotonin, ghrelin, and peptide YY (PYY) were significantly decreased in patients with PCOS compared with controls, and have a significantly negative correlation with waist circumference and testosterone. Sequencing of the V3–V4 region of the 16S rRNA gene in fecal samples revealed the substantial differences of gut microbial species between the PCOS and non-obese controls. Bacterial species were clustered into 23 co-abundance groups (CAGs) based on the SparCC correlation coefficients of their relative abundance. The CAGs increased in PCOS, including the bacteria belonging to *Bacteroides, Escherichia/Shigella* and *Streptococcus*, were negatively correlated with ghrelin, and positively correlated with testosterone and BMI. Furthermore, the CAGs that were decreased in PCOS, including the bacteria from *Akkermansia* and Ruminococcaceae, showed opposite relationship with body-weight, sex-hormone, and brain–gut peptides. In conclusion, gut microbial dysbiosis in women with PCOS is associated with the disease phenotypes.

## Introduction

Polycystic ovary syndrome is a common heterogeneous endocrine and chronic metabolic disease affecting 3–26% of reproductive-aged women by applying the different diagnostic criteria (Michelmore et al., 1999; March et al., 2010; Li et al., 2013). Women with PCOS are faced with increased risk of endometrial cancer, diabetes, metabolic syndrome and cardiovascular disease in the long term (Legro et al., 1999; Hardiman et al., 2003; Dumesic and Lobo, 2013). They showed hyperandrogenism, hyperinsulinemia, anovulation, and polycystic ovarian morphology (Landay et al., 2009), and had 2.8-fold higher prevalence of obesity compared to women without PCOS according to a recent meta-analysis (Lim et al., 2013). Although the genetic (Vink et al., 2006), gestational environment (Abbott et al., 1998) and lifestyle factors (Zhang J. et al., 2015) have been suggested to involve in the development of PCOS, the precise underlying triggers for these key biochemical and metabolic disturbances remain largely unclear.

Since high levels of circulating insulin stimulating the ovarian theca cells to produce androgen had been supported by various experiments, both *in vitro* and *in vivo* (Poretsky, 1991), a new microbiological hypothesis for the development of PCOS suggested that dysbiosis of gut microbiota can increase the ovaries production of androgens, and then interfere with normal follicle development via triggering a chronic inflammatory response and insulin resistance (Tremellen and Pearce, 2012). Further evidence suggests that gut microbiota and its metabolites have the ability to regulate inflammation pathway activation, brain–gut peptide secretion and islet β-cell proliferation, thus leading to abnormal or excessive fat accumulation, insulin resistance and compensatory hyperinsulinemia (Vrieze et al., 2010; Barber et al., 2015). The shifts of gut microbiota had been observed in the letrozole-induced PCOS mouse models (Guo et al., 2016; Kelley et al., 2016), and fecal microbiota transplantation from the letrozole-induced PCOS mice to germ-free mice indicated that gut microbiota also has a close relationship with its host’s sex hormone levels, estruscycles and ovarian morphological changes (Guo et al., 2016). In addition, according to another mouse model experiment, female recipients of male cecal microbiota displayed increased testosterone levels, compared with unmanipulated females and female recipients of female cecal micriobiota (Markle et al., 2013). On the other hand, some mediators of the brain–gut axis, such as serotonin, ghrelin and PYY, whose secretion were affected by gut microbiota (Samuel et al., 2008; Yano et al., 2015; Perry et al., 2016), were involved in appetite regulation, systemic energy homeostasis and LH secretion (Konturek et al., 2004; Ogata et al., 2009; Jun et al., 2015). But the experimental evidence of the relationship between gut microbiota, mediators of the brain–gut axis and metabolic phenotypes of PCOS in human is still absent.

In order to study the association of gut microbiota with obesity and non-obesity in relation to PCOS, we recruited non-obese or obese women with or without PCOS. We investigated the composition of gut microbiota in PCOS, and how the changes of gut microbiota correlated with the sex-hormones, brain–gut peptides, and various other metabolic parameters. Our study suggested an altered composition of gut microbiota of women with PCOS from controls, and identified important bacterial phylotypes which were associated with the clinical characteristics of PCOS.

## Materials and Methods

### Study Participants

A total of 48 premenopausal women aged 17–45 years were enrolled from individuals who visited the department of endocrinology or gynecology at Shanghai General Hospital (Shanghai, China) between January 2014 and July 2014. Diagnosis criteria for PCOS established by Chinese Medical Association was based on Rotterdam criteria and the currently available evidences in Han Chinese. In our study, to avoid the confusion due to the different diagnostic criteria, each participant had met the three features based on Rotterdam criteria and the Chinese criteria as follows: oligo- or anovulation, clinical and/or biochemical signs of hyperandrogenism, and polycystic ovaries (Rotterdam Eshre/Asrm-Sponsored Pcos Consensus Workshop Group, 2004). Women in control group had no history of diagnosed PCOS and did not meet any of the Rotterdam Criteria. Individuals with one of the followings were excluded from the study: pregnancy, androgen-secreting tumors, adrenal disorders, thyroid dysfunction, Cushing’s syndrome, smoking, hypertension, and lipid-dysregulation. Included participants had also no administration of hormonal medication, insulin sensitizer or antibiotics within the preceding 3 months. After submitting written informed consent, all participants with PCOS (*n* = 33) or without PCOS (*n* = 15) were then stratified according to BMI into the following four subgroups: obese PCOS patients (PO group, *n* = 21), non-obese PCOS patients (PN group, *n* = 12), obese controls (CO group, *n* = 6) and non-obese controls (CN group, *n* = 9). Obesity was defined as BMI ≥ 25 kg/m^2^ and the non-obesity otherwise according to the World Health Organization (World Health Organization, 2000). The study protocol was approved by the Human Research Ethics Committee of Shanghai General Hospital (No. 2014KY091) ahead of procedure of enrollment. The trial was registered in Chinese Clinical Trial Registry with the registration number ChiCTR-TRC-14005075.

### Sampling

All subjects were examined in the morning after overnight fasting (≥8 h). 4-(2-aminoethyl)-benzenesulfonyl fluoride was immediately added to the blood samples for enzyme-linked immunosorbent assay (ELISA) of ghrelin at a final concentration of 1 mg/mL. After clotting at room temperature for 30 min, the blood samples were centrifuged at 3,000 ×*g* for 15 min at 4°C, and were transferred into separate tubes and acidified by adding 5 N HCl to a final concentration of 0.05 N. The processed serum samples were stored at -20°C until analysis. For PYY, blood samples were immediately added DPP-IV inhibitor and aprotinin to a final concentration 500 KIU/mL. Fecal samples were collected on the same day. Feces was divided into aliquots and was frozen on dry ice immediately upon collection and stored at -80°C until analysis.

### Anthropometric and Metabolic Parameters Measurements

Waist circumference was measured midway between the lower costal margin and the iliac crest, and hip circumference at the level of maximum extension of the buttocks. WHR was calculated as waist circumference divided by hip circumference. BMI was calculated as body weight in kilograms divided by body height squared in meters.

Biochemical indexes were measured on automatic biochemical analyzer (Beckman AU5800 Clinical Chemistry System, Beckman Coulter, Inc., South Kraemer Boulevard, Brea, CA, USA). HbA1c level was measured by high-performance liquid chromatography (Bio-Rad Variant II Turbo, Bio-Rad Laboratories, Co., Ltd, Germany). FINS, FSH, LH, and testosterone were tested using an automated immunoassay system (TOSOHAIA-1800ST, TOSOH Corporation, Tokyo, Japan). The HOMA-IR and beta cell function (HOMA-beta) were calculated using the following formula:

(1) HOMA-IR = fasting plasma glucose (FPG) (mM) * FINS (mIU/L)/22.5;

(2) HOMA-beta = 20 * FINS (mIU/l)/(FPG (mM) -3.5)*100%.

Plasma serotonin, ghrelin, and PYY levels were determined with the commercially available ELISA kit according to manufacturer’s instructions. The kit for serotonin was purchased from EUROIMMUNUS, Inc. (EA602/96, Mountain Lakes, NJ, USA), and kits for ghrelin and PYY were bought from EMD Millipore Corporation (EZGRT-89K, EZHPYYT66K, Saint Charles, MO, USA, respectively). The intra-assay and inter-assay coefficients of variation were <5 and <10, respectively.

The investigation of mental psychological health was conducted with SDS and SAS.

### Fecal DNA Extraction and Sequencing

DNA extraction from each frozen fecal sample was conducted as previously described (Godon et al., 1997). All samples were sequenced on the Illumina Miseq platform (Illumina, Inc., USA) with Miseq reagent kit v3 (600-cycle) (MS-102-3033, Illumina, USA). Sequencing library of the V3–V4 regions of the 16S rRNA gene was constructed based on the manufacturer’s instruction (Part # 15044223 Rev. B, Illumina, USA), with some modifications as previously published (Zhang et al., 2016).

### Statistical Analysis

#### Clinical Data Statistical Analysis

Statistical analyses were performed using SAS statistical software 9.2 (SAS Institute, Inc., Cary, NC, USA). Normal distribution of the data was calculated with the Kolmogorov–Smirnov test. Continuous variables were expressed as mean ± standard deviation (SD). Variables that were not normally distributed were rank transformed before analysis. Differences on clinical characteristics and metabolic biomarkers were evaluated using *t*-test or a one-way analysis of variance (ANOVA) for normally distributed variables or non-parameter Wilcoxon test for non-normally distributed, with multiple comparisons performed using SNK (Student-Newman-Keuls) correction. Partial correlation analysis was used to study the relationships between brain–gut peptides and other metabolic parameters. A multiple stepwise regression analysis was employed to identify influence factors of brain–gut peptides, using a stepwise selection method with a *P-*value of 0.15 at entry and a *P*-value of 0.15 at stay. All independent variables in the multiple regression were tested for multicollinearity. Statistical tests were based on two tailed probability. A *P*-value less than 0.05 was considered statistically significant. Power calculation was performed using PASS software 11.

#### Bioinformatics and Statistical Analysis

Quality control of the raw sequencing data was performed, as previously described (Zhang et al., 2016), to reduce spurious OTUs. OTUs were clustered and mapped using Uparse (Edgar, 2013) according to Uparse OTU analysis pipeline^1^. Quality-filtered sequences were clustered into unique sequences and sorted in order of decreasing abundance to identify representative sequences, and singletons were omitted in this step. Chimera detection was performed using UCHIME (Edgar et al., 2011) against the Ribosomal Database Project (RDP) classifier (Cole et al., 2013). OTUs were clustered using a ≥97% similarity threshold. The representative sequences of each OTU were used to generate a phylogenetic tree using FastTree (Price et al., 2009), and were subjected to RDP for taxonomical assignments with a bootstrap cutoff of 80%.

The sequences of all the samples were downsized to 7500 (1000 permutations) to equal the difference in sequencing depth. The rarefaction estimates, the Shannon index, the Simpson’s diversity index and the Chao1 index were calculated using QIIME v1.2.1 (Caporaso et al., 2010), and the difference of alpha-diversity was evaluated by Kruskal–Wallis test using IBM SPSS Statistics 19. To evaluate the structural segregation of gut microbiota between individuals with/without PCOS, we performed CAP (Anderson and Willis, 2003) using FORTRAN program (Anderson, 2004). And MANOVA was estimated using MATLAB R2014a (The MathWorks, Natick, MA, USA). Both of CAP and MANOVA analysis were calculated based on the Bray–Curtis (Bray and Curtis, 1957) and the UniFrac distance (Lozupone and Knight, 2005) performed by QIIME.

To pick key OTUs that might be responsible for the segregation, RDA was performed using the R ‘vegan’ package. Statistical significance was assessed by Monte Carlo test with 9999 permutations.

The OTUs shared by at least 20% among all the samples were considered as prevalent OTUs. Correlations between prevalent OTUs were calculated using the SparCC algorithm (Friedman and Alm, 2012) based on their abundance. The correlation values were converted to a correlation distance (1-correlation value), and the OTUs were clustered using the Ward clustering algorithm via MATLAB. Permutational MANOVA was used to determine CAGs via dividing the cluster tree (9999 permutations, *P* < 0.001). Permutational MANOVA was performed using the R ‘vegan’ package^2^ and the CAG network was visualized in Cytoscape. Spearman correlation between CAGs and clinical parameters was calculated using MATLAB, and Benjamini-Hochberg method was used to control false discovery rate (Benjamini and Hochberg, 1995). Kruskal–Wallis test was performed for comparing the difference of relative abundance at CAG level between the four groups using IBM SPSS Statistics 19.

Raw sequencing data of the 16S rRNA gene V3–V4 regions and accompanying information are available in Sequence Read Archive database under accession number SRP085887.

## Results

### General Anthropometric and Metabolic Parameters of All the Participants

The clinical data of all the 48 participants was summarized in **Table 1**. After adjusted *P* value for age, the differences between groups reflected in the following aspects: anthropometric parameters, sex hormones, metabolic parameters, mediators of the brain–gut axis and psychological scales. Compared to women without PCOS, women with PCOS showed significantly higher levels of testosterone and hirsutism score. PO group also had increased levels of metabolic parameters and SDS value than PN group, indicating that excess weight aggravated the metabolic abnormalities in women with PCOS. The level of serotonin was significantly higher in CN group than the other three groups. The level of ghrelin was lower in women with PCOS compared with women without PCOS, and it was also lower in obese women with PCOS compared with non-obese women with PCOS. PYY showed a significant decrease in PO group compared with CN and CO groups, while PN group was also decreased compared to CN and CO and it was higher than the PO group. Both PCOS patients and obese individuals possessed abnormal levels of the mediators of the brain–gut axis.

**Table 1:** The clinical characteristics and metabolic profiles of all the participants.

Parameters	Controls (*n* = 15)		PCOS (*n* = 33)			
	Non-obese, *n* = 9	Obese, *n* = 6	Non-obese, *n* = 12	Obese, *n* = 21	*F*	*P* adjusted for age
Age (years)	32.2 ± 5.9	33 ± 5.4	25.5 ± 4.3	29.3 ± 6.5	3.3	/
**Anthropometric parameters**				
BMI (kg/m^2^)	21.9 ± 2.2^b^	27.5 ± 3.3^a^	21.6 ± 2.2^b^	30 ± 3.6^a^	46	<0.0001
Waist circumference (cm)	68.8 ± 5.7^c^	84.7 ± 12.3^b^	75 ± 7.8^c^	95.1 ± 9.2^a^	24	<0.0001
WHR	0.78 ± 0.05^c^	0.8 ± 0.1^b^	0.79 ± 0.06^bc^	0.92 ± 0.05^a^	20	<0.0001
Hirsutism score	8 ± 0^b^	8 ± 0^b^	17.5 ± 6.5^a^	16.6 ± 5.7^a^	31	<0.0001
**Blood RT**				
Leukocyte (10^9/L)	6.8 ± 2	6.4 ± 1	7.1 ± 2.1	7.3 ± 1.7	0.5	0.49
Neutrocyte (10^9/L)	4.1 ± 0.8	4.1 ± 1.2	4.5 ± 2	4.6 ± 1.2	0.5	0.6638
Lymphocyte (10^9/L)	2.1 ± 0.9	2.3 ± 0.6	2 ± 0.4	2.3 ± 0.7	0.4	0.7929
**Sex hormones**				
FSH (IU/L)	5.3 ± 2.2	7 ± 0.9	7.2 ± 2.3	6.4 ± 2.5	1.6	0.2072
LH (IU/L)	7.5 ± 4.1^b^	11.3 ± 5.7^ab^	15.7 ± 8.5^a^	19.4 ± 31.2^ab^	3.2	0.0335
Testosterone (nmol/L)	0.79 ± 0.41^b^	1 ± 0.4^b^	4.5 ± 2^a^	5.4 ± 2.7^a^	14	<0.0001
LH/FSH	1.5 ± 0.7	1.6 ± 0.7	2.3 ± 1.1	2.5 ± 2	2.1	0.1117
**Metabolic parameters**				
FPG (mmol/L)	5 ± 0.4	5.2 ± 0.4	4.7 ± 0.5	5 ± 2.1	2.7	0.0591
PPG (mmol/L)	6 ± 0.9^b^	5.5 ± 1^b^	6 ± 1.3^b^	8.5 ± 2.6^a^	8.8	0.0001
FINS (uU/mL)	11.9 ± 4^a^	15.6 ± 5^a^	5.5 ± 2.4^b^	14.3 ± 7.4^a^	11	<0.0001
P2hINS (uU/mL)	54.1 ± 31.5^b^	83.7 ± 52.4^ab^	65.5 ± 53.3^b^	163 ± 99.3^a^	7.7	0.0003
HOMA-IR	1.7 ± 1.1^a^	3.5 ± 1^a^	1.1 ± 0.5^b^	3.3 ± 2.1^a^	11	<0.0001
HOMA-beta	159.8 ± 32.7^a^	203.7 ± 107^a^	174.8 ± 284.2^a^	339.8 ± 254.8^a^	3.3	0.0279
HbA1c (%)	5 ± 0.3^b^	5.3 ± 0.3^b^	5.2 ± 0.3^b^	5.9 ± 1.1^a^	9.4	<0.0001
ALT (U/L)	14 ± 9.6^b^	16.2 ± 10.7^b^	17.5 ± 8.9^ab^	32.9 ± 28.3^a^	4.1	0.0123
AST (U/L)	15.9 ± 1.9	16.5 ± 5.4	19.4 ± 5.5	27.7 ± 23.6	2.1	0.1198
GGT (U/L)	14.1 ± 3.6^b^	19.2 ± 10.4^ab^	13.8 ± 3.4^b^	26.3 ± 23.6^a^	4.8	0.0057
TCH (mmol/L)	4.5 ± 0.9	4.8 ± 0.9	4.4 ± 1.1	4.6 ± 0.7	0.2	0.8762
TG (mmol/L)	1 ± 0.6^b^	1.3 ± 1^ab^	0.83 ± 0.38^b^	1.5 ± 0.7^a^	7.1	0.0005
**Mediators of the brain–gut axis**				
Serotonin (ng/mL)	294.2 ± 91.8^a^	199.6 ± 38.1^b^	211.5 ± 69^b^	193.6 ± 54.4^b^	4.1	0.012
Ghrelin (ng/mL)	0.51 ± 0.08^a^	0.44 ± 0.04^ab^	0.4 ± 0.1^b^	0.24 ± 0.05^c^	27	<0.0001
PYY (pg/mL)	79.8 ± 24.1^a^	79.2 ± 17.5^a^	56.8 ± 16.1^ab^	62.1 ± 48.4^b^	4.9	0.0052
**Psychological scales**				
SDS value	39.2 ± 8.1^b^	42.4 ± 12.4^ab^	36.9 ± 6.9^b^	50.1 ± 9.4^a^	6.6	0.0009
SAS value	32.7 ± 5.5^b^	34.2 ± 7.2^ab^	35.9 ± 4.5^ab^	42.8 ± 9.1^a^	5.4	0.003

*Data are presented as mean ± SD. Multiple comparison: means with the same letter (a, b, or c) are not significantly different, with different letters are significantly different (adjusted *P* < 0.05). *P*-value by ANOVA for normally distributed variables or non-parameter Wilcoxon test for non-normally distributed. The parameters of age, waist circumference, leukocyte, testosterone, AST, ALT, TCH, and SDS value were normally distributed. PCOS, polycystic ovary syndrome; BMI, body mass index; WHR, Waist hip ratio; Blood RT, blood routine test; FSH, follicular stimulating hormone; LH, luteinizing hormone; FPG, fasting plasma glucose; PPG, 2h postprandial plasma glucose; FINS, fasting plasma insulin; P2hINS, 2h postprandial plasma insulin; HOMA-IR, Homeostasis model assessment for insulin resistance index; HOMA-beta, Homeostasis model assessment for beta cell function; HbA1c, Hemoglobin A1c; ALT, alanine aminotransferase; AST, aspartate transaminase; GGT, γ-glutamyltransferase; TCH, total cholesterol; TG, triglyceride; PYY, peptide YY; SDS, self-rating depression scale; SAS, self-rating anxiety scale.*

In order to evaluate the association between the mediators of the brain–gut axis and the various metabolic characteristics of PCOS, we calculated the partial correlation between serotonin, PYY, ghrelin and the clinical parameters of all subjects after adjusting for age and BMI (**Table 2**). Plasma serotonin level was significantly negatively correlated with waist circumference, hirsutism score, testosterone and SAS value, whereas, significantly positively correlated with neutrocyte count, HOMA-beta and TCH. Plasma PYY level was significantly negatively correlated with waist circumference, WHR, hirsutism score, testosterone, PPG, ALT and AST, whereas, significantly positively correlated with neutrocyte count and HOMA-beta. Plasma ghrelin level was significantly negatively correlated with waist circumference, WHR, hirsutism score, testosterone, PPG, P2hINS, ALT, and AST. To explore whether plasma brain–gut peptide/indole levels were independently associated with the clinical parameters, multiple stepwise regression analysis involving all the parameters with significant correlations with the mediators of the brain–gut axis was performed (with sle = 0.15, and sls = 0.15). Adjusting for age and BMI, it revealed that the testosterone level (β, -2.5296; *P* < 0.001), neutrocyte count (β, 0.38753; *P* < 0.01) and waist circumference (β, -0.29091; *P* < 0.05) were independently associated with plasma serotonin levels; the waist circumference (β, -0.27497; *P* < 0.05), the PCOS group (β, -9.447; *P* < 0.05) and neutrocyte count (β, 0.41872; *P* < 0.01) were independently associated with plasma PYY levels; while the testosterone level (β, -1.3728; *P* < 0.05), the PCOS group (β, -12.6121; *P* < 0.001), BMI (β, -0.889; *P* < 0.01), and P2hINS (β, -0.3382; *P* < 0.01) were independently associated with plasma ghrelin levels in all participants. Thus, the levels of the brain–gut peptides and the brain–gut indole were correlated with the PCOS related phenotypes.

**Table 2:** Partial correlations between the brain–gut peptide/indole levels and various clinical parameters after adjusted for age and BMI.

Parameters	Serotonin		PYY		Ghrelin	
	*r*	*P*	*r*	*P*	*r*	*P*
**Anthropometric parameters**				
Waist circumference	**-0.331**	**0.025**	**-0.507**	**0.000**	**-0.297**	**0.045**
WHR	**-**0.279	0.061	**-0.379**	**0.010**	**-0.336**	**0.023**
Hirsutism score	**-0.340**	**0.026**	**-0.421**	**0.005**	**-0.381**	**0.012**
**Blood RT**				
Leukocyte count	0.265	0.082	0.164	0.287	**-**0.008	0.959
Neutrocyte count	**0.322**	**0.033**	**0.350**	**0.020**	**-**0.062	0.689
Lymphocyte count	0.205	0.181	0.028	0.856	0.059	0.706
**Sex hormones**				
FSH	0.053	0.726	0.237	0.113	**-**0.072	0.636
LH	**-**0.127	0.399	**-**0.136	0.369	**-**0.222	0.139
Testosterone	**-0.312**	**0.035**	**-0.371**	**0.011**	**-0.627**	**<0.0001**
LH/FSH	**-**0.221	0.140	**-**0.255	0.087	**-**0.187	0.213
**Metabolic parameters**				
FPG	**-**0.157	0.298	**-**0.065	0.669	**-**0.012	0.939
PPG	**-**0.047	0.757	**-0.396**	**0.007**	**-0.418**	**0.004**
FINS	0.173	0.251	0.083	0.583	0.086	0.568
P2hINS	0.062	0.682	**-**0.024	0.874	**-0.338**	**0.022**
HOMA-beta	**0.351**	**0.017**	**0.317**	**0.032**	0.157	0.298
HbA1c	0.024	0.874	**-**0.164	0.276	**-**0.111	0.462
ALT	0.014	0.926	**-0.291**	**0.050**	**-0.418**	**0.004**
AST	0.042	0.784	**-0.303**	**0.041**	**-0.301**	**0.042**
TCH	**0.312**	**0.035**	**-**0.156	0.299	**-**0.061	0.688
TG	0.115	0.447	**-**0.253	0.090	**-**0.186	0.215
**Mediators of the brain–gut axis**				
Serotonin	1.000	/	0.282	0.058	0.215	0.151
Ghrelin	0.215	0.151	0.238	0.112	1.000	/
PYY	0.282	0.058	1.000	/	0.238	0.112
**Psychological scales**				
SDS value	**-**0.185	0.219	**-**0.135	0.370	**-**0.034	0.825
SAS value	**-0.355**	**0.016**	**-**0.231	0.122	**-**0.064	0.671

*After taking account of age and BMI, bold values indicate statistical significance determined by Spearman partial correlation analysis. BMI, body mass index; WHR, Waist hip ratio; Blood RT, blood routine test; FSH, follicular stimulating hormone; LH, luteinizing hormone; FPG, fasting plasma glucose; PPG, 2h postprandial plasma glucose; FINS, fasting plasma insulin; P2hINS, 2h postprandial plasma insulin; HOMA-beta, homeostasis model assessment for beta cell function; HbA1c, hemoglobin A1c; ALT, alanine aminotransferase; AST, aspartate transaminase; TCH, total cholesterol; TG, triglyceride; PYY, peptide YY; SDS, self-rating depression scale; SAS, self-rating anxiety scale; CN, control with non-obesity; CO, control with obesity; PN, PCOS with non-obesity; PO, PCOS with obesity.*

### Dysbiosis of Gut Microbiota in Women with PCOS

**FIGURE 1:**
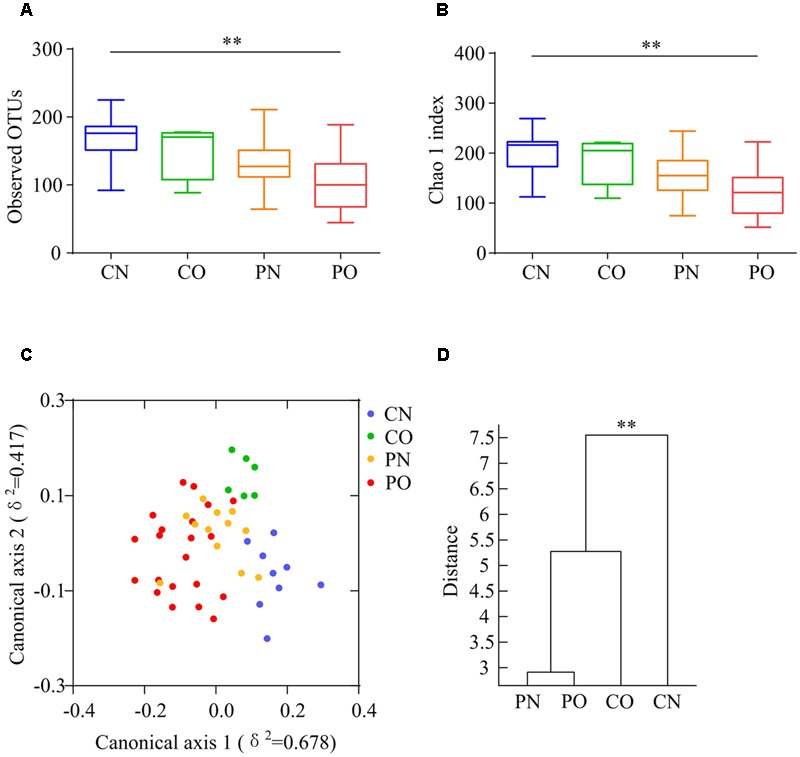
**Overall structural differentiation of gut microbiota based on UniFrac distance between four groups. (A)** OTU-level rarefaction (observed OTUs). **(B)** Chao 1 index. Values are shown by box-plot. Box represents the interquartile range. The line inside the box represents the median. And whiskers denote the minimum and maximum value. ^∗∗^Adjusted *P* < 0.01 (Kruskal–Wallis test). **(C)** Bray–Curtis CAP using the first 13 PCs (accounting for 80.67% of the total variation). **(D)** Clustering of gut microbiota based on Bray–Curtis distance calculated with MANOVA using the first 13 PCs. ^∗∗^*P* < 0.01. CN: non-obese control group, *n* = 9. CO: obese control group, *n* = 6. PN: non-obese PCOS group, *n* = 12. PO: obese PCOS group, *n* = 21.

We performed gene sequencing on the V3–V4 regions of the 16S rRNA to evaluate the dysbiosis of gut microbiota in PCOS. In total, 852,886 usable raw reads (264,105 unique sequences) were obtained from all 48 samples. 770,892 high-quality reads (14,480 ± 4,325 reads per sample) were clustered into 567 OTUs at 97% similarity level. According to observed OTUs and the Chao1 index, CN group owned the highest richness, followed by CO and PN group, and PO group showed the lowest richness of gut microbiota (**Figures 1A,B**), while no difference was found for the Shannon index and the Simpson’s diversity index. CAP and MANOVA based on the Bray–Curtis distance revealed a separation between CN group and the other three groups (**Figures 1C,D**). Notably, the gut microbiota composition of CO group was more similar with that of PN and PO groups, rather than CN group. CAP and MANOVA based on the UniFrac distance also showed similar trends (Supplementary Figure S1). Thus, the overall composition of the gut microbiota was disrupted in PCOS.

**FIGURE 2:**
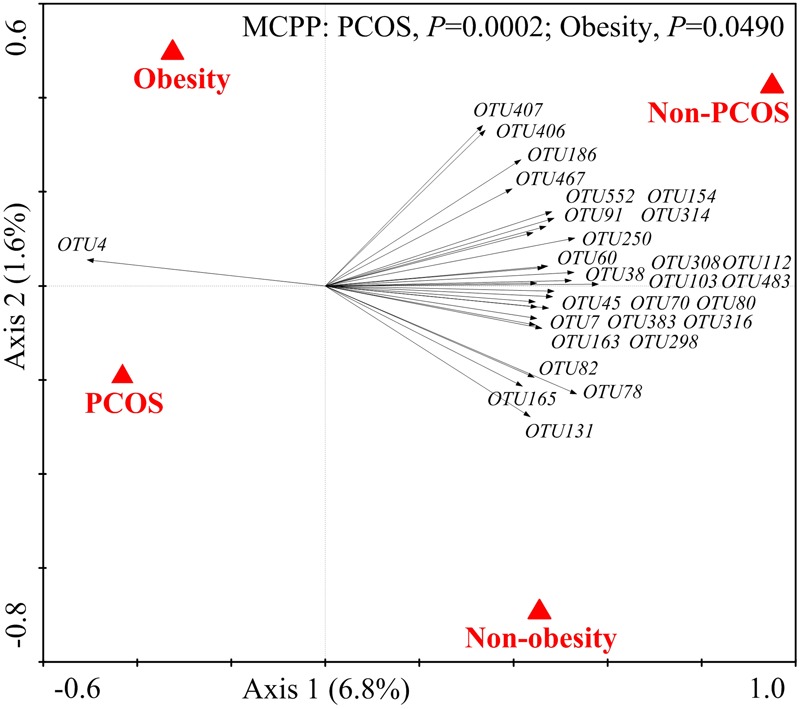
**The 28 key OTUs selected by RDA responding to PCOS or obesity.** PCOS and obesity were conducted as environmental variables. Relative abundances (after log transformation) of all OTUs were used as response variables, and responding OTUs that had at least 20% of the variability explained by all canonical axes were selected and exhibited by black arrows. Statistical significance was assessed by Monte Carlo test with 9999 permutations.

**FIGURE 3:**
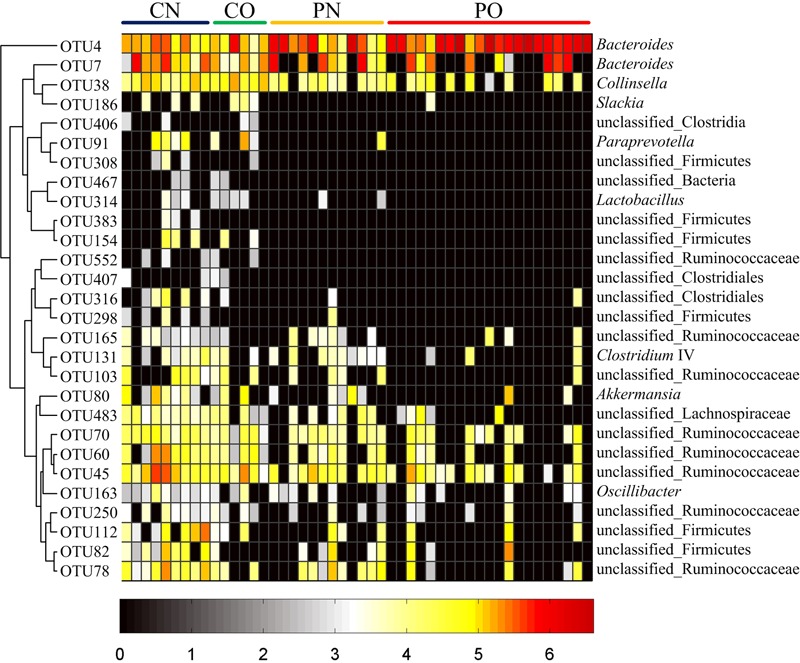
**Heat map of the relative abundances of 28 key OTUs related to the alteration of gut microbiota between the four groups.** The color of the spots represents the relative abundance (normalized and log-transformed) of the OTUs. OTUs are ordered by spearman correlation analysis based on relative abundance. The genus-level taxonomic assignment is shown on the right. For CN, *n* = 9; for PN, *n* = 12; for CO, *n* = 6; and for PO, *n* = 21.

We then used RDA to identify the gut bacteria responding to PCOS and obesity. RDA confirmed that both PCOS and obesity were significantly correlated to the dysbiosis of gut microbiota (Monte Carlo Permutation Procedure, *P* = 0.0002 and 0.0490, respectively). We identified 28 key OTUs that responded to the differentiation between the four groups according to RDA (**Figure 2**). OTU4 belonging to genus *Bacteroides* was enriched in PO group than CN and CO groups (*P* < 0.01 and *P* < 0.05, respectively), with PN group falling in between them. The average relative abundances of 21 OTUs were higher in CN group than PO group (*P* < 0.05), which mostly belonged to the genera *Akkermansia, Bacteroides, Clostridium* IV, *Lactobacillus, Oscillibacter*, or unclassified genera from family Ruminococcaceae, while in CO and PN groups, the abundances of these OTUs were lower than CN group but were higher than PO group. Five OTUs were enriched in CO group, which belonged to the genera *Collinsella, Paraprevotella, Slackia*, and unclassified genera from class Clostridia and kingdom Bacteria. OTU7 belonging to *Bacteroides* showed decreased abundance in CO group, compared with the other three groups (**Figure 3**).

### Association between Gut Microbiota and Clinical Parameters in PCOS and Control Subjects

**FIGURE 4:**
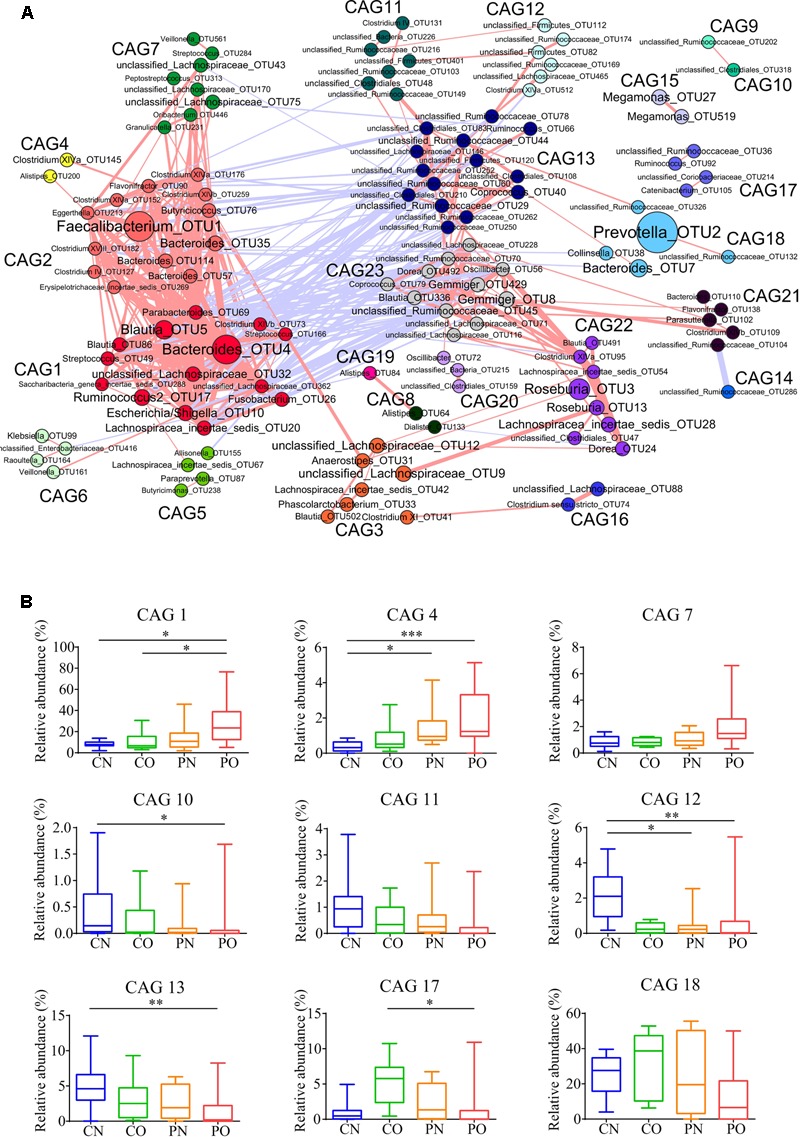
**Bacterial correlation based on relative abundance in women with or without PCOS. (A)** Co-abundance groups interaction network. Network shows correlation relationships between 23 CAGs of 225 OTUs from all samples. Node size represents the average abundance of each OTU. Lines between nodes represent correlations of each other, with the line width representing the correlation magnitude. The red ones represent positive correlations, and the blue ones represent negative correlations. Only lines whose absolute value of correlation coefficient greater than 0.50 are drawn, and unconnected nodes are omitted. **(B)** Group-level abundance differentiation of CAGs. Data are visualized by box-plot. Box represents the interquartile range. The line inside the box represents the median. And whiskers denote the minimum and maximum value. ^∗^adjusted *P* < 0.05, ^∗∗^adjusted *P* < 0.01, and ^∗∗∗^adjusted *P* < 0.001 (multiple comparisons of Kruskal–Wallis test). For CN, *n* = 9; For PN, *n* = 12; For CO, *n* = 6; For PO, *n* = 21.

As the bacteria act as interdependent functional groups (guilds) in the gut ecosystem (Zhang C. et al., 2015), we constructed a co-abundance network at OTU level. There were 225 OTUs shared by at least 20% of the samples were clustered into 23 CAGs based on SparCC correlation coefficients (**Figure 4A**; Supplementary Table S1). The results showed bacteria species in the same genus could have different response to the environment perturbations. For example, OTU5, 86, 506, 393, 491, and 336 belonging to genus *Blautia* were divided into CAG1 (both OTU5 and 506), 3, 15, 22, and 23, respectively. According to the Kruskal–Wallis test, 14 CAGs showed no significant difference between the four groups. CAG1, 4, and 7 were enriched in PO group rather than CN group. CAG 1 contained 14 OTUs, which belonged to genera *Bacteroides, Escherichia/Shigella, Streptococcus, Blautia*, and *Parabacteroides*. CAG4 contained 5 OTUs, which mainly belonged to *Clostridium* XlVa, *Alistipes* and *Weissella*. CAG7 contained OTUs belonging to *Granulicatella, Streptococcus, Peptostreptococcus*, and *Rothia*. While, CAG10, 11, 12, and 13 decreased in PO group rather than CN group. The dominant OTUs in CAG10-13 mostly belonged to *Akkermansia, Alistipes, Coprococcus*, and *Ruminococcus*. In CO and PN groups, the relative abundances of all these above CAGs fell in between those in PO and CN groups. In addition, CAG17 and 18 showed increased trends in CO group compared with the other groups (**Figure 4B**).

**FIGURE 5:**
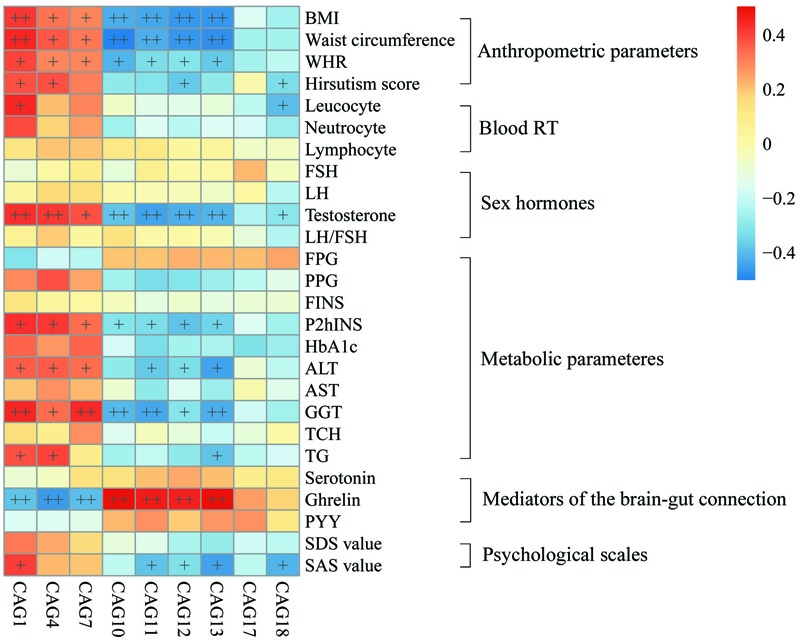
**Associations between clinical parameters and gut microbiota.** The color of spots represents R value of Spearman correlation between each CAG and clinical parameter. +FDR < 0.05, ++FDR < 0.01. For leucocyte, neutrocyte, lymphocyte and hirsutism, *n* = 46; for the other parameters, *n* = 48. BMI, body mass index; WHR, Waist hip ratio; Blood RT, blood routine test; FSH, follicular stimulating hormone; LH, luteinizing hormone; FPG, fasting plasma glucose; PPG, 2h postprandial plasma glucose; FINS, fasting plasma insulin; P2hINS, 2h postprandial plasma insulin; HOMA-IR, homeostasis model assessment for insulin resistance index; HOMA-beta, homeostasis model assessment for beta cell function; HbA1c, hemoglobin A1c; ALT, alanine aminotransferase; AST, aspartate transaminase; GGT, γ-glutamyltransferase; TCH, total cholesterol; TG, triglyceride; PYY, peptide YY; SDS, self-rating depression scale; SAS, self-rating anxiety scale.

To explore the relationship between gut microbiota and host phenotype, Spearman correlation between 27 clinical parameters and 9 CAGs that showed significant difference among the four groups (**Figure 5**). The results showed significant correlation between gut microbiota and PCOS related symptoms, including obesity, inflammation, hyperinsulinmia, hyperandrogenism, abnormal ghrelin level, and psychological state. CAG1, 4 and 7, which were enriched in PO group, had a positive correlation with the disease phenotypes; while CAG10–13 and 18, which were enriched in CN group, had an opposite trend.

## Discussion

Our current study showed a broad view of the association between PCOS and gut microbiota in human. Our study, to the best of our knowledge, is the first clinical study that revealed the overall composition of gut microbiota in women with PCOS. In this study, we observed an altered gut microbial pattern in both obese and non-obese women with PCOS against non-obese control individuals, and found that the gut microbiota of PCOS shares similar compositional dysbiosis with that of obesity. Furthermore, the altered gut microbiota of PCOS was associated with the metabolic parameters, the sex-hormones and the mediators of the brain–gut axis.

Polycystic ovary syndrome leads to several complications, which include hyperandrogenism, obesity, and metabolic syndrome. In the current work, compared to women with obesity or PCOS alone, obese women with PCOS had more deleterious metabolic disorder, including insulin resistance and dyslipidemia, in accord with previous studies (Lim et al., 2013; Behboudigandevani et al., 2016). Based on our data, changes of gut microbiota also had trends similar to the variations of metabolic symptoms. Women with obesity and PCOS shared similar dysbiosis of gut microbiota. When compared with women with obesity or PCOS alone, obese women with PCOS possessed a more serious dysbiosis of gut microbiota. We observed that some gram-negative bacteria belonging to the genera *Bacteroides* and *Escherichia/Shigella* significantly increased in the gut of PCOS women with obesity. Bacterial LPS produced by gram-negative bacteria has been demonstrate to induce chronic inflammation, obesity, and insulin resistance in LPS-infused mice (Cani et al., 2007). While various experiments, both *in vitro* and *in vivo*, have verified that high levels of circulating insulin stimulates the ovarian theca cells to produce androgen (Poretsky, 1991). It suggests that gut microbiota antigen might also associate to the metabolic disorder in PCOS. On the contrary, we found that *Akkermensia* decreased in obesity and PCOS, which can restore host mucus layer and has been considered to contribute to the reduction in metabolic endotoxemia (Everard et al., 2013). Hence, the dysbiosis of gut microbiota, as increasing LPS-producing bacteria and decreasing protective bacteria, may correlate with the development of metabolic disorder in PCOS.

Some mediators of the brain–gut axis, such as serotonin and ghrelin were involved in appetite regulation (Konturek et al., 2004; Jun et al., 2015) as well as psychological well-being in PCOS (Lang et al., 2015). Moreover, intracerebroventricular injection of ghrelin significantly reduced serum LH level and pulsatile frequency in rats (Ogata et al., 2009). However, the relationship between the levels of serum mediators of the brain–gut axis and PCOS remains controversial in human studies. For example, the ghrelin level had significant difference between PCOS and BMI-matched controls in a Spanish study (Micic et al., 2007), but this orexigenic hormone showed association only with body weight in an Australian PCOS cohort (Samantha et al., 2014). The fasting PYY level also had conflicting results from different cohort (Zwirska-Korczala et al., 2008; Lin et al., 2015). Our study showed that women with PCOS had a significant decrease of serotonin, ghrelin, and PYY level compared with controls, and obesity enhanced the difference of serotonin and ghrelin in PCOS. Moreover, we find that the levels of serotonin, PYY, and ghrelin had a negative correlation with PCOS related parameters, such as waist circumference and testosterone. Our results indicated that the mediators of the brain–gut axis are associated with PCOS.

The secretion of some mediators of the brain–gut axis is regulated by gut microbiota. It has been shown that serotonin biosynthesis is promoted by spore-forming bacteria isolated from the mouse and human gut microbiota, known to be dominated by Clostridial species, while species belonging to *Bacteroides* didn’t show this function (Yano et al., 2015). In our study, most of the decreased key OTUs were Clostridial species, and the increased key OTU was *Bacteroides* spp. in both PCOS and obesity cohorts. PYY secretion is influenced by short chain fatty acid (SCFA) produced by gut microbiota (Tremaroli and Bäckhed, 2012). In the current, we also found the association between ghrelin and gut microbiota. For example, CAG1, which includes *Bacteroides, Escherichia/Shigella* and *Blautia*, was negatively correlated with ghrelin, while CAG10, including *Akkermansia*, was positively correlated with ghrelin. But we did not find a significant association between serotonin, PYY and the gut microbiota, which might be explained by the limitation of small sample size and require further studies. Regardless, according to existing evidences, regulating the mediators of the brain–gut axis might be one of the pathways for gut microbiota to effect on PCOS.

This cross-sectional study revealed the association between gut microbiota and PCOS. The dysbiosis of gut microbiota might contribute to the development of PCOS through the possible mechanisms discussed above. Due to the limited sample size (power calculation: the power values for BMI, waist circumference, testosterone level and fasting insulin level were 99.0, 96.9, 89.5, and 70.4% respectively, α = 0.0083) and the observation of association but not causality, further studies are needed to enlarge sample size and explore the potential causal mechanisms between gut microbiota and PCOS, such as the metabolites produced by gut microbiota and fecal microbiota transplantation.

Taken together, our work suggested an altered composition of gut microbiota, represented by the reduction of alpha diversity, the increase of LPS-producing bacteria, and the decrease of spore-forming species. This study also demonstrated the association between gut microbiota and PCOS-related clinical parameters. The previous study did not find any association of the saliva microbiota and PCOS phenotypes (Lindheim et al., 2016), indicating that the gut microbiota has a closer relationship with PCOS. It laid a foundation of research on the interaction between gut microbiota and its host with PCOS.

## Ethics Statement

This study was carried out in accordance with the recommendations of Guideline on Informed Consent, Human Research Ethics Committee of Shanghai General Hospital with written informed consent from all subjects. All subjects gave written informed consent in accordance with the Declaration of Helsinki. The protocol was approved by the Human Research Ethics Committee of Shanghai General Hospital.

## Author Contributions

The authors XD, YP, CZ, and RL conceived the study and designed the experiments. YS, JS, FZ, LL, XW, YL, HF, and WD performed the technical procedures. RL and XD provided the interpretation of data and wrote the paper. AG, AR, LZ, and XD revised the final draft of the paper. All authors read and approved the final version of the paper.

## Conflict of Interest Statement

The authors declare that the research was conducted in the absence of any commercial or financial relationships that could be construed as a potential conflict of interest.

## Footnotes

1 http://drive5.com/usearch/manual/uparse_pipeline.html

2 http://www.r-project.org/

## Abbreviations

ALT: alanine aminotransferase
AST: aspartate transaminase
Blood RT: blood routine test
BMI: body mass index
CAG: co-abundance group
CAP: canonical analysis of principal coordinates
CN: control with non-obesity
CO: control with obesity
FINS: fasting plasma insulin
FSH: follicular stimulating hormone
FPG: fasting plasma glucose
GGT: γ-glutamyltransferase
HbA1c: hemoglobin A1c
HOMA-beta: homeostasis model assessment for beta cell function
HOMA-IR: homeostasis model assessment for insulin resistance index
LH: luteinizing hormone
LPS: lipopolysaccharide
MANOVA: multivariate analysis of variance
OTU: operational taxonomic units
P2hINS: 2h postprandial plasma insulin
PCOS: polycystic ovary syndrome
PN: PCOS with non-obesity
PO: PCOS with obesity
PPG: 2h postprandial plasma glucose
PYY: peptide YY
RDA: redundancy analysis
SAS: self-rating anxiety scale
SDS: self-rating depression scale
TCH: total cholesterol
TG: triglyceride
WHR: waist hip ratio
