# Supplementary material for: Dysbiosis of Gut Microbiota Associated with Clinical Parameters in Polycystic Ovary Syndrome
Source: Front Microbiol. 2017 Feb 28;8:324. doi: 10.3389/fmicb.2017.00324 (PMC5328957; doi:10.3389/fmicb.2017.00324)
Supplement: Supplementary file 1 [file Data_Sheet_1.DOCX]

**Supplementary Information**

**
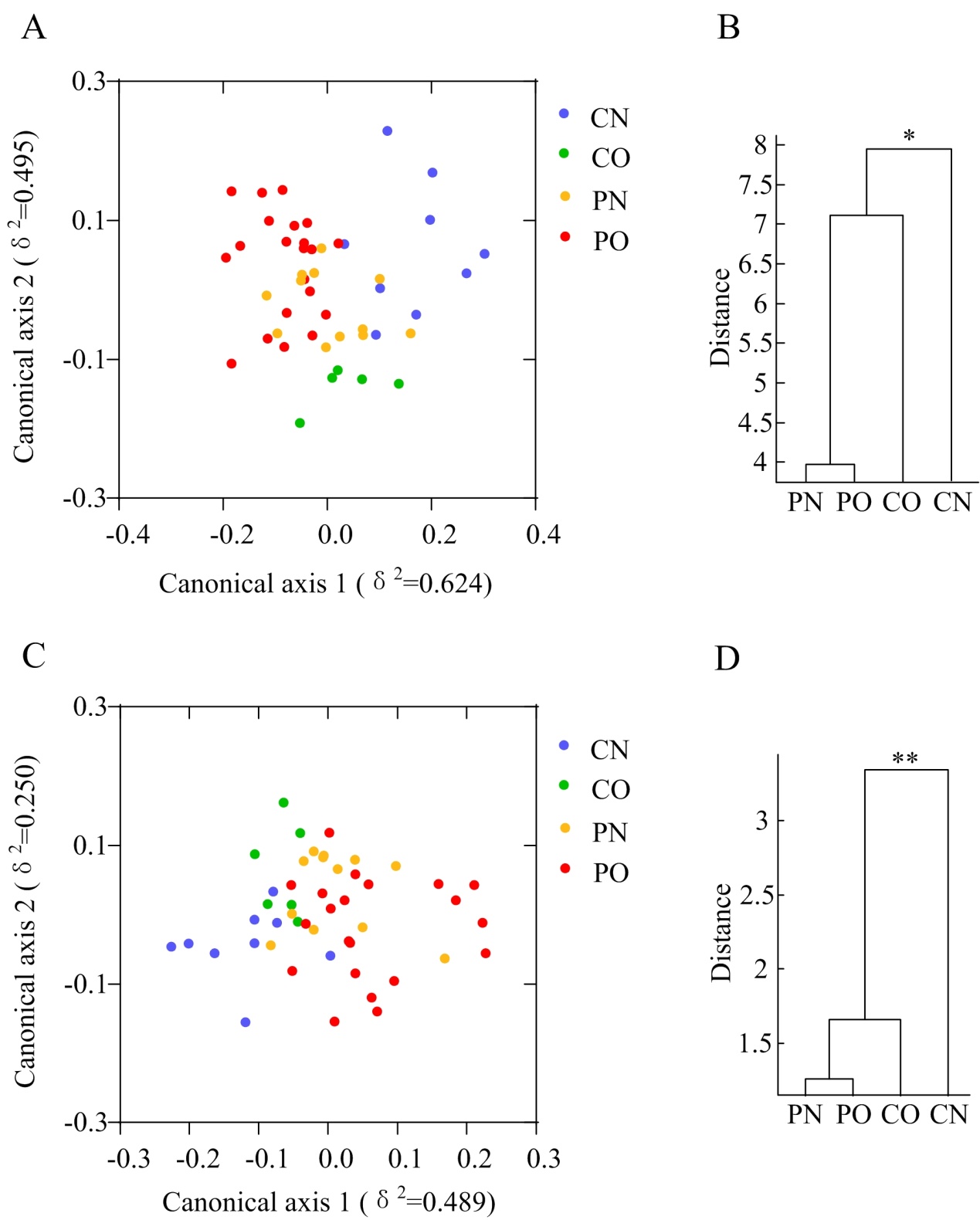
**

**Supplementary Figure 1: Structural differentiation of gut microbiota based on UniFrac distance.** (A) Unweighted-UniFrac CAP using the first 17 PCs (accounting for 81.13% of the total variation). (B) Clustering of gut microbiota based on unweighted-UniFrac distance calculated with MANOVA using the first 17 PCs. (C) Weighted-UniFrac CAP using the first 7 PCs (accounting for 80.49% of the total variation). (D) Clustering of gut microbiota based on Weighted-UniFrac distance calculated with MANOVA using the first 7 PCs. **P*<0.05, ***P*<0.01. CN: Non-obese control group, n=9. CO: Obese control group, n=6. PN: Non-obese PCOS group, n=12. PO: Obese PCOS group, n=21.

**Supplementary Table 1:** **Taxonomical information of the 225 predominant OTUs in 23 CAGs.**

| **OTU_ID** | **CAG** | **Taxonomical assignments** | | | | | |
| --- | --- | --- | --- | --- | --- | --- | --- |
|  |  | **Domain** | **Phylum** | **Class** | **Order** | **Famliy** | **Genus** |
| OTU32 | CAG1 | Bacteria | Firmicutes | Clostridia | Clostridiales | Lachnospiraceae |  |
| OTU69 | CAG1 | Bacteria | Bacteroidetes | Bacteroidia | Bacteroidales | Porphyromonadaceae | Parabacteroides |
| OTU4 | CAG1 | Bacteria | Bacteroidetes | Bacteroidia | Bacteroidales | Bacteroidaceae | Bacteroides |
| OTU362 | CAG1 | Bacteria | Firmicutes | Clostridia | Clostridiales | Lachnospiraceae |  |
| OTU5 | CAG1 | Bacteria | Firmicutes | Clostridia | Clostridiales | Lachnospiraceae | Blautia |
| OTU20 | CAG1 | Bacteria | Firmicutes | Clostridia | Clostridiales | Lachnospiraceae | Lachnospiracea_incertae_sedis |
| OTU86 | CAG1 | Bacteria | Firmicutes | Clostridia | Clostridiales | Lachnospiraceae | Blautia |
| OTU26 | CAG1 | Bacteria | Fusobacteria | Fusobacteriia | Fusobacteriales | Fusobacteriaceae | Fusobacterium |
| OTU10 | CAG1 | Bacteria | Proteobacteria | Gammaproteobacteria | Enterobacteriales | Enterobacteriaceae | Escherichia/Shigella |
| OTU166 | CAG1 | Bacteria | Firmicutes | Bacilli | Lactobacillales | Streptococcaceae | Streptococcus |
| OTU49 | CAG1 | Bacteria | Firmicutes | Bacilli | Lactobacillales | Streptococcaceae | Streptococcus |
| OTU288 | CAG1 | Bacteria | Candidatus Saccharibacteria | Saccharibacteria_genera_incertae_sedis | Saccharibacteria_genera_incertae_sedis | Saccharibacteria_genera_incertae_sedis | Saccharibacteria_genera_incertae_sedis |
| OTU17 | CAG1 | Bacteria | Firmicutes | Clostridia | Clostridiales | Lachnospiraceae | Ruminococcus2 |
| OTU73 | CAG1 | Bacteria | Firmicutes | Clostridia | Clostridiales | Lachnospiraceae | Clostridium XlVb |
| OTU1 | CAG2 | Bacteria | Firmicutes | Clostridia | Clostridiales | Ruminococcaceae | Faecalibacterium |
| OTU269 | CAG2 | Bacteria | Firmicutes | Erysipelotrichia | Erysipelotrichales | Erysipelotrichaceae | Erysipelotrichaceae_incertae_sedis |
| OTU182 | CAG2 | Bacteria | Firmicutes | Erysipelotrichia | Erysipelotrichales | Erysipelotrichaceae | Clostridium XVIII |
| OTU259 | CAG2 | Bacteria | Firmicutes | Clostridia | Clostridiales | Lachnospiraceae | Clostridium XlVb |
| OTU114 | CAG2 | Bacteria | Bacteroidetes | Bacteroidia | Bacteroidales | Bacteroidaceae | Bacteroides |
| OTU213 | CAG2 | Bacteria | Actinobacteria | Actinobacteria | Coriobacteriales | Coriobacteriaceae | Eggerthella |
| OTU35 | CAG2 | Bacteria | Bacteroidetes | Bacteroidia | Bacteroidales | Bacteroidaceae | Bacteroides |
| OTU76 | CAG2 | Bacteria | Firmicutes | Clostridia | Clostridiales | Ruminococcaceae | Butyricicoccus |
| OTU127 | CAG2 | Bacteria | Firmicutes | Clostridia | Clostridiales | Ruminococcaceae | Clostridium IV |
| OTU152 | CAG2 | Bacteria | Firmicutes | Clostridia | Clostridiales | Lachnospiraceae | Clostridium XlVa |
| OTU57 | CAG2 | Bacteria | Bacteroidetes | Bacteroidia | Bacteroidales | Bacteroidaceae | Bacteroides |
| OTU176 | CAG2 | Bacteria | Firmicutes | Clostridia | Clostridiales | Lachnospiraceae | Clostridium XlVa |
| OTU90 | CAG2 | Bacteria | Firmicutes | Clostridia | Clostridiales | Ruminococcaceae | Flavonifractor |
| OTU11 | CAG3 | Bacteria | Bacteroidetes | Bacteroidia | Bacteroidales | Bacteroidaceae | Bacteroides |
| OTU25 | CAG3 | Bacteria | Actinobacteria | Actinobacteria | Bifidobacteriales | Bifidobacteriaceae | Bifidobacterium |
| OTU18 | CAG3 | Bacteria | Bacteroidetes | Bacteroidia | Bacteroidales | Bacteroidaceae | Bacteroides |
| OTU41 | CAG3 | Bacteria | Firmicutes | Clostridia | Clostridiales | Peptostreptococcaceae | Clostridium XI |
| OTU39 | CAG3 | Bacteria | Actinobacteria | Actinobacteria | Bifidobacteriales | Bifidobacteriaceae | Bifidobacterium |
| OTU496 | CAG3 | Bacteria | Bacteroidetes | Bacteroidia | Bacteroidales | Bacteroidaceae | Bacteroides |
| OTU12 | CAG3 | Bacteria | Firmicutes | Clostridia | Clostridiales | Lachnospiraceae |  |
| OTU502 | CAG3 | Bacteria | Firmicutes | Clostridia | Clostridiales | Lachnospiraceae | Blautia |
| OTU9 | CAG3 | Bacteria | Firmicutes | Clostridia | Clostridiales | Lachnospiraceae |  |
| OTU193 | CAG3 | Bacteria | Firmicutes | Clostridia | Clostridiales | Lachnospiraceae |  |
| OTU31 | CAG3 | Bacteria | Firmicutes | Clostridia | Clostridiales | Lachnospiraceae | Anaerostipes |
| OTU33 | CAG3 | Bacteria | Firmicutes | Negativicutes | Selenomonadales | Acidaminococcaceae | Phascolarctobacterium |
| OTU42 | CAG3 | Bacteria | Firmicutes | Clostridia | Clostridiales | Lachnospiraceae | Lachnospiracea_incertae_sedis |
| OTU145 | CAG4 | Bacteria | Firmicutes | Clostridia | Clostridiales | Lachnospiraceae | Clostridium XlVa |
| OTU21 | CAG4 | Bacteria | Firmicutes | Clostridia | Clostridiales | Lachnospiraceae | Clostridium XlVa |
| OTU200 | CAG4 | Bacteria | Bacteroidetes | Bacteroidia | Bacteroidales | Rikenellaceae | Alistipes |
| OTU175 | CAG4 | Bacteria | Firmicutes | Bacilli | Lactobacillales | Leuconostocaceae | Weissella |
| OTU201 | CAG4 | Bacteria | Proteobacteria | Betaproteobacteria | Burkholderiales |  |  |
| OTU155 | CAG5 | Bacteria | Firmicutes | Negativicutes | Selenomonadales | Veillonellaceae | Allisonella |
| OTU67 | CAG5 | Bacteria | Firmicutes | Clostridia | Clostridiales | Lachnospiraceae | Lachnospiracea_incertae_sedis |
| OTU334 | CAG5 | Bacteria | Cyanobacteria/Chloroplast | Chloroplast | Chloroplast | Chloroplast | Streptophyta |
| OTU96 | CAG5 | Bacteria | Firmicutes | Clostridia | Clostridiales | Clostridiaceae 1 | Clostridium sensu stricto |
| OTU238 | CAG5 | Bacteria | Bacteroidetes | Bacteroidia | Bacteroidales | Porphyromonadaceae | Butyricimonas |
| OTU87 | CAG5 | Bacteria | Bacteroidetes | Bacteroidia | Bacteroidales | Prevotellaceae | Paraprevotella |
| OTU276 | CAG5 | Bacteria | Firmicutes | Clostridia | Clostridiales | Ruminococcaceae |  |
| OTU81 | CAG5 | Bacteria | Firmicutes | Negativicutes | Selenomonadales | Veillonellaceae | Megasphaera |
| OTU323 | CAG5 | Bacteria | Firmicutes | Clostridia | Clostridiales | Lachnospiraceae |  |
| OTU15 | CAG6 | Bacteria | Firmicutes | Negativicutes | Selenomonadales | Veillonellaceae | Dialister |
| OTU161 | CAG6 | Bacteria | Firmicutes | Negativicutes | Selenomonadales | Veillonellaceae | Veillonella |
| OTU164 | CAG6 | Bacteria | Proteobacteria | Gammaproteobacteria | Enterobacteriales | Enterobacteriaceae | Raoultella |
| OTU416 | CAG6 | Bacteria | Proteobacteria | Gammaproteobacteria | Enterobacteriales | Enterobacteriaceae |  |
| OTU62 | CAG6 | Bacteria | Firmicutes | Clostridia | Clostridiales | Peptostreptococcaceae | Clostridium XI |
| OTU254 | CAG6 | Bacteria | Firmicutes | Bacilli | Lactobacillales | Enterococcaceae | Enterococcus |
| OTU99 | CAG6 | Bacteria | Proteobacteria | Gammaproteobacteria | Enterobacteriales | Enterobacteriaceae | Klebsiella |
| OTU170 | CAG7 | Bacteria | Firmicutes | Clostridia | Clostridiales | Lachnospiraceae |  |
| OTU43 | CAG7 | Bacteria | Firmicutes | Clostridia | Clostridiales | Lachnospiraceae |  |
| OTU231 | CAG7 | Bacteria | Firmicutes | Bacilli | Lactobacillales | Carnobacteriaceae | Granulicatella |
| OTU446 | CAG7 | Bacteria | Firmicutes | Clostridia | Clostridiales | Lachnospiraceae | Oribacterium |
| OTU313 | CAG7 | Bacteria | Firmicutes | Clostridia | Clostridiales | Peptostreptococcaceae | Peptostreptococcus |
| OTU371 | CAG7 | Bacteria | Actinobacteria | Actinobacteria | Actinomycetales | Micrococcaceae | Rothia |
| OTU381 | CAG7 | Bacteria | Proteobacteria | Gammaproteobacteria | Pasteurellales | Pasteurellaceae |  |
| OTU257 | CAG7 | Bacteria | Firmicutes | Bacilli | Lactobacillales | Streptococcaceae | Streptococcus |
| OTU469 | CAG7 | Bacteria | Actinobacteria | Actinobacteria | Actinomycetales | Actinomycetaceae | Actinomyces |
| OTU284 | CAG7 | Bacteria | Firmicutes | Bacilli | Lactobacillales | Streptococcaceae | Streptococcus |
| OTU561 | CAG7 | Bacteria | Firmicutes | Negativicutes | Selenomonadales | Veillonellaceae | Veillonella |
| OTU526 | CAG7 | Bacteria | Firmicutes | Bacilli | Lactobacillales | Streptococcaceae | Streptococcus |
| OTU75 | CAG7 | Bacteria | Firmicutes | Clostridia | Clostridiales | Lachnospiraceae |  |
| OTU125 | CAG8 | Bacteria | Firmicutes | Clostridia | Clostridiales | Ruminococcaceae |  |
| OTU197 | CAG8 | Bacteria | Firmicutes | Clostridia | Clostridiales | Ruminococcaceae | Anaerotruncus |
| OTU229 | CAG8 | Bacteria | Bacteroidetes | Bacteroidia | Bacteroidales | Bacteroidaceae | Bacteroides |
| OTU133 | CAG8 | Bacteria | Firmicutes | Negativicutes | Selenomonadales | Veillonellaceae | Dialister |
| OTU317 | CAG8 | Bacteria | Bacteroidetes | Bacteroidia | Bacteroidales | Rikenellaceae | Alistipes |
| OTU225 | CAG8 | Bacteria | Bacteroidetes | Bacteroidia | Bacteroidales | Rikenellaceae | Alistipes |
| OTU227 | CAG8 | Bacteria | Firmicutes | Clostridia | Clostridiales | Ruminococcaceae |  |
| OTU342 | CAG8 | Bacteria | Bacteroidetes | Bacteroidia | Bacteroidales | Porphyromonadaceae | Parabacteroides |
| OTU171 | CAG8 | Bacteria | Bacteroidetes | Bacteroidia | Bacteroidales | Porphyromonadaceae | |
| OTU64 | CAG8 | Bacteria | Bacteroidetes | Bacteroidia | Bacteroidales | Rikenellaceae | Alistipes |
| OTU94 | CAG8 | Bacteria | Proteobacteria | Deltaproteobacteria | Desulfovibrionales | Desulfovibrionaceae | Bilophila |
| OTU178 | CAG8 | Bacteria | Firmicutes | Clostridia | Clostridiales | Ruminococcaceae | Flavonifractor |
| OTU237 | CAG8 | Bacteria | Firmicutes | Erysipelotrichia | Erysipelotrichales | Erysipelotrichaceae | Holdemania |
| OTU158 | CAG9 | Bacteria | Firmicutes | Clostridia | Clostridiales | Ruminococcaceae |  |
| OTU320 | CAG9 | Bacteria | Bacteroidetes | Bacteroidia | Bacteroidales | Porphyromonadaceae | Butyricimonas |
| OTU331 | CAG9 | Bacteria | Firmicutes | Clostridia | Clostridiales | Ruminococcaceae |  |
| OTU260 | CAG9 | Bacteria |  |  |  |  |  |
| OTU339 | CAG9 | Bacteria | Firmicutes | Clostridia | Clostridiales | Ruminococcaceae |  |
| OTU202 | CAG9 | Bacteria | Firmicutes | Clostridia | Clostridiales | Ruminococcaceae |  |
| OTU318 | CAG10 | Bacteria | Firmicutes | Clostridia | Clostridiales |  |  |
| OTU80 | CAG10 | Bacteria | Verrucomicrobia | Verrucomicrobiae | Verrucomicrobiales | Verrucomicrobiaceae | Akkermansia |
| OTU272 | CAG10 | Bacteria | Firmicutes |  |  |  |  |
| OTU281 | CAG10 | Bacteria | Actinobacteria | Actinobacteria | Coriobacteriales | Coriobacteriaceae |  |
| OTU397 | CAG10 | Bacteria | Firmicutes | Clostridia | Clostridiales |  |  |
| OTU236 | CAG10 | Bacteria | Firmicutes | Clostridia | Clostridiales | Ruminococcaceae | Clostridium IV |
| OTU303 | CAG10 | Bacteria | Firmicutes |  |  |  |  |
| OTU300 | CAG10 | Bacteria | Firmicutes | Clostridia | Clostridiales | Ruminococcaceae |  |
| OTU398 | CAG10 | Bacteria | Firmicutes | Clostridia | Clostridiales | Ruminococcaceae |  |
| OTU328 | CAG10 | Bacteria | Firmicutes | Clostridia | Clostridiales |  |  |
| OTU100 | CAG11 | Bacteria | Firmicutes |  |  |  |  |
| OTU103 | CAG11 | Bacteria | Firmicutes | Clostridia | Clostridiales | Ruminococcaceae |  |
| OTU216 | CAG11 | Bacteria | Firmicutes | Clostridia | Clostridiales | Ruminococcaceae |  |
| OTU226 | CAG11 | Bacteria |  |  |  |  |  |
| OTU124 | CAG11 | Bacteria | Firmicutes |  |  |  |  |
| OTU401 | CAG11 | Bacteria | Firmicutes |  |  |  |  |
| OTU149 | CAG11 | Bacteria | Firmicutes | Clostridia | Clostridiales | Ruminococcaceae |  |
| OTU131 | CAG11 | Bacteria | Firmicutes | Clostridia | Clostridiales | Ruminococcaceae | Clostridium IV |
| OTU460 | CAG11 | Bacteria | Firmicutes | Clostridia | Clostridiales | Lachnospiraceae |  |
| OTU48 | CAG11 | Bacteria | Firmicutes | Clostridia | Clostridiales |  |  |
| OTU112 | CAG12 | Bacteria | Firmicutes |  |  |  |  |
| OTU169 | CAG12 | Bacteria | Firmicutes | Clostridia | Clostridiales | Ruminococcaceae |  |
| OTU174 | CAG12 | Bacteria | Firmicutes | Clostridia | Clostridiales | Ruminococcaceae |  |
| OTU82 | CAG12 | Bacteria | Firmicutes |  |  |  |  |
| OTU130 | CAG12 | Bacteria | Bacteroidetes | Bacteroidia | Bacteroidales | Rikenellaceae | Alistipes |
| OTU218 | CAG12 | Bacteria |  |  |  |  |  |
| OTU505 | CAG12 | Bacteria | Firmicutes | Clostridia | Clostridiales |  |  |
| OTU163 | CAG12 | Bacteria | Firmicutes | Clostridia | Clostridiales | Ruminococcaceae | Oscillibacter |
| OTU184 | CAG12 | Bacteria | Actinobacteria | Actinobacteria | Coriobacteriales | Coriobacteriaceae |  |
| OTU165 | CAG12 | Bacteria | Firmicutes | Clostridia | Clostridiales | Ruminococcaceae |  |
| OTU465 | CAG12 | Bacteria | Firmicutes | Clostridia | Clostridiales | Lachnospiraceae |  |
| OTU483 | CAG12 | Bacteria | Firmicutes | Clostridia | Clostridiales | Lachnospiraceae |  |
| OTU512 | CAG12 | Bacteria | Firmicutes | Clostridia | Clostridiales | Lachnospiraceae | Clostridium XlVa |
| OTU531 | CAG12 | Bacteria | Firmicutes | Clostridia | Clostridiales | Lachnospiraceae | Coprococcus |
| OTU108 | CAG13 | Bacteria | Firmicutes | Clostridia | Clostridiales |  |  |
| OTU250 | CAG13 | Bacteria | Firmicutes | Clostridia | Clostridiales | Ruminococcaceae |  |
| OTU146 | CAG13 | Bacteria | Firmicutes | Clostridia | Clostridiales | Lachnospiraceae |  |
| OTU66 | CAG13 | Bacteria | Firmicutes | Clostridia | Clostridiales | Ruminococcaceae | Ruminococcus |
| OTU252 | CAG13 | Bacteria | Firmicutes | Clostridia | Clostridiales | Ruminococcaceae |  |
| OTU83 | CAG13 | Bacteria | Firmicutes | Clostridia | Clostridiales |  |  |
| OTU262 | CAG13 | Bacteria | Firmicutes | Clostridia | Clostridiales | Ruminococcaceae |  |
| OTU44 | CAG13 | Bacteria | Firmicutes | Clostridia | Clostridiales | Ruminococcaceae |  |
| OTU40 | CAG13 | Bacteria | Firmicutes | Clostridia | Clostridiales | Lachnospiraceae | Coprococcus |
| OTU60 | CAG13 | Bacteria | Firmicutes | Clostridia | Clostridiales | Ruminococcaceae |  |
| OTU120 | CAG13 | Bacteria | Firmicutes |  |  |  |  |
| OTU29 | CAG13 | Bacteria | Firmicutes | Clostridia | Clostridiales | Ruminococcaceae |  |
| OTU210 | CAG13 | Bacteria | Firmicutes | Clostridia | Clostridiales |  |  |
| OTU78 | CAG13 | Bacteria | Firmicutes | Clostridia | Clostridiales | Ruminococcaceae |  |
| OTU101 | CAG14 | Bacteria | Proteobacteria | Betaproteobacteria | Burkholderiales | Sutterellaceae | Sutterella |
| OTU6 | CAG14 | Bacteria | Bacteroidetes | Bacteroidia | Bacteroidales | Bacteroidaceae | Bacteroides |
| OTU119 | CAG14 | Bacteria | Bacteroidetes | Bacteroidia | Bacteroidales | Bacteroidaceae | Bacteroides |
| OTU183 | CAG14 | Bacteria | Firmicutes | Clostridia | Clostridiales | Ruminococcaceae |  |
| OTU286 | CAG14 | Bacteria | Firmicutes | Clostridia | Clostridiales | Ruminococcaceae |  |
| OTU61 | CAG14 | Bacteria | Firmicutes | Clostridia | Clostridiales |  |  |
| OTU196 | CAG14 | Bacteria | Lentisphaerae | Lentisphaeria | Victivallales | Victivallaceae | Victivallis |
| OTU302 | CAG14 | Bacteria | Firmicutes | Clostridia | Clostridiales | Ruminococcaceae | Oscillibacter |
| OTU296 | CAG14 | Bacteria | Firmicutes | Clostridia | Clostridiales |  |  |
| OTU128 | CAG15 | Bacteria | Firmicutes | Clostridia | Clostridiales | Ruminococcaceae | Clostridium IV |
| OTU141 | CAG15 | Bacteria | Firmicutes | Clostridia | Clostridiales | Lachnospiraceae |  |
| OTU208 | CAG15 | Bacteria | Firmicutes | Clostridia | Clostridiales | Lachnospiraceae | Clostridium XlVa |
| OTU55 | CAG15 | Bacteria | Bacteroidetes | Bacteroidia | Bacteroidales | Porphyromonadaceae | |
| OTU27 | CAG15 | Bacteria | Firmicutes | Negativicutes | Selenomonadales | Veillonellaceae | Megamonas |
| OTU519 | CAG15 | Bacteria | Firmicutes | Negativicutes | Selenomonadales | Veillonellaceae | Megamonas |
| OTU135 | CAG15 | Bacteria | Bacteroidetes | Bacteroidia | Bacteroidales | Porphyromonadaceae | Parabacteroides |
| OTU50 | CAG15 | Bacteria | Firmicutes | Clostridia | Clostridiales |  |  |
| OTU151 | CAG15 | Bacteria | Actinobacteria | Actinobacteria | Coriobacteriales | Coriobacteriaceae |  |
| OTU295 | CAG15 | Bacteria | Firmicutes | Clostridia | Clostridiales | Lachnospiraceae |  |
| OTU393 | CAG15 | Bacteria | Firmicutes | Clostridia | Clostridiales | Lachnospiraceae | Blautia |
| OTU191 | CAG16 | Bacteria | Firmicutes | Erysipelotrichia | Erysipelotrichales | Erysipelotrichaceae | Turicibacter |
| OTU97 | CAG16 | Bacteria | Firmicutes | Clostridia | Clostridiales | Ruminococcaceae | Butyricicoccus |
| OTU74 | CAG16 | Bacteria | Firmicutes | Clostridia | Clostridiales | Clostridiaceae 1 | Clostridium sensu stricto |
| OTU88 | CAG16 | Bacteria | Firmicutes | Clostridia | Clostridiales | Lachnospiraceae |  |
| OTU105 | CAG17 | Bacteria | Firmicutes | Erysipelotrichia | Erysipelotrichales | Erysipelotrichaceae | Catenibacterium |
| OTU214 | CAG17 | Bacteria | Actinobacteria | Actinobacteria | Coriobacteriales | Coriobacteriaceae |  |
| OTU37 | CAG17 | Bacteria | Bacteroidetes | Bacteroidia | Bacteroidales | Prevotellaceae | Prevotella |
| OTU36 | CAG17 | Bacteria | Firmicutes | Clostridia | Clostridiales | Ruminococcaceae |  |
| OTU92 | CAG17 | Bacteria | Firmicutes | Clostridia | Clostridiales | Ruminococcaceae | Ruminococcus |
| OTU58 | CAG17 | Bacteria | Proteobacteria | Betaproteobacteria | Burkholderiales | Sutterellaceae | Sutterella |
| OTU93 | CAG17 | Bacteria | Proteobacteria | Gammaproteobacteria | Pasteurellales | Pasteurellaceae | Haemophilus |
| OTU132 | CAG18 | Bacteria | Firmicutes | Clostridia | Clostridiales | Ruminococcaceae |  |
| OTU2 | CAG18 | Bacteria | Bacteroidetes | Bacteroidia | Bacteroidales | Prevotellaceae | Prevotella |
| OTU14 | CAG18 | Bacteria | Firmicutes | Clostridia | Clostridiales | Lachnospiraceae | Lachnospiracea_incertae_sedis |
| OTU247 | CAG18 | Bacteria | Firmicutes | Clostridia | Clostridiales | Lachnospiraceae | Lachnospiracea_incertae_sedis |
| OTU326 | CAG18 | Bacteria | Firmicutes | Clostridia | Clostridiales | Ruminococcaceae |  |
| OTU258 | CAG18 | Bacteria | Firmicutes | Clostridia | Clostridiales | Lachnospiraceae |  |
| OTU38 | CAG18 | Bacteria | Actinobacteria | Actinobacteria | Coriobacteriales | Coriobacteriaceae | Collinsella |
| OTU7 | CAG18 | Bacteria | Bacteroidetes | Bacteroidia | Bacteroidales | Bacteroidaceae | Bacteroides |
| OTU52 | CAG18 | Bacteria | Firmicutes | Erysipelotrichia | Erysipelotrichales | Erysipelotrichaceae | Erysipelotrichaceae_incertae_sedis |
| OTU63 | CAG18 | Bacteria | Bacteroidetes | Bacteroidia | Bacteroidales | Bacteroidaceae | Bacteroides |
| OTU106 | CAG19 | Bacteria | Bacteroidetes | Bacteroidia | Bacteroidales | Porphyromonadaceae | Barnesiella |
| OTU168 | CAG19 | Bacteria | Bacteroidetes | Bacteroidia | Bacteroidales | Porphyromonadaceae | Butyricimonas |
| OTU121 | CAG19 | Bacteria | Bacteroidetes | Bacteroidia | Bacteroidales | Rikenellaceae | Alistipes |
| OTU177 | CAG19 | Bacteria | Bacteroidetes | Bacteroidia | Bacteroidales | Porphyromonadaceae | Butyricimonas |
| OTU192 | CAG19 | Bacteria | Firmicutes | Clostridia | Clostridiales |  |  |
| OTU84 | CAG19 | Bacteria | Bacteroidetes | Bacteroidia | Bacteroidales | Rikenellaceae | Alistipes |
| OTU159 | CAG20 | Bacteria | Firmicutes | Clostridia | Clostridiales |  |  |
| OTU215 | CAG20 | Bacteria |  |  |  |  |  |
| OTU53 | CAG20 | Bacteria | Firmicutes | Clostridia | Clostridiales | Ruminococcaceae | Clostridium IV |
| OTU72 | CAG20 | Bacteria | Firmicutes | Clostridia | Clostridiales | Ruminococcaceae | Oscillibacter |
| OTU19 | CAG20 | Bacteria | Firmicutes | Clostridia | Clostridiales | Ruminococcaceae | Ruminococcus |
| OTU22 | CAG20 | Bacteria | Bacteroidetes | Bacteroidia | Bacteroidales | Rikenellaceae | Alistipes |
| OTU23 | CAG20 | Bacteria | Bacteroidetes | Bacteroidia | Bacteroidales | Porphyromonadaceae | Parabacteroides |
| OTU242 | CAG20 | Bacteria | Firmicutes | Clostridia | Clostridiales | Lachnospiraceae | Clostridium XlVb |
| OTU379 | CAG20 | Bacteria | Firmicutes | Clostridia | Clostridiales | Ruminococcaceae | Oscillibacter |
| OTU102 | CAG21 | Bacteria | Proteobacteria | Betaproteobacteria | Burkholderiales | Sutterellaceae | Parasutterella |
| OTU109 | CAG21 | Bacteria | Firmicutes | Clostridia | Clostridiales | Lachnospiraceae | Clostridium XlVb |
| OTU110 | CAG21 | Bacteria | Bacteroidetes | Bacteroidia | Bacteroidales | Bacteroidaceae | Bacteroides |
| OTU138 | CAG21 | Bacteria | Firmicutes | Clostridia | Clostridiales | Ruminococcaceae | Flavonifractor |
| OTU59 | CAG21 | Bacteria | Bacteroidetes | Bacteroidia | Bacteroidales | Porphyromonadaceae | Odoribacter |
| OTU104 | CAG21 | Bacteria | Firmicutes | Clostridia | Clostridiales | Ruminococcaceae |  |
| OTU34 | CAG21 | Bacteria | Firmicutes | Erysipelotrichia | Erysipelotrichales | Erysipelotrichaceae | Clostridium XVIII |
| OTU13 | CAG22 | Bacteria | Firmicutes | Clostridia | Clostridiales | Lachnospiraceae | Roseburia |
| OTU491 | CAG22 | Bacteria | Firmicutes | Clostridia | Clostridiales | Lachnospiraceae | Blautia |
| OTU68 | CAG22 | Bacteria | Firmicutes | Clostridia | Clostridiales | Lachnospiraceae | Lachnospiracea_incertae_sedis |
| OTU3 | CAG22 | Bacteria | Firmicutes | Clostridia | Clostridiales | Lachnospiraceae | Roseburia |
| OTU47 | CAG22 | Bacteria | Firmicutes | Clostridia | Clostridiales |  |  |
| OTU173 | CAG22 | Bacteria | Firmicutes | Clostridia | Clostridiales | Ruminococcaceae |  |
| OTU28 | CAG22 | Bacteria | Firmicutes | Clostridia | Clostridiales | Lachnospiraceae | Lachnospiracea_incertae_sedis |
| OTU24 | CAG22 | Bacteria | Firmicutes | Clostridia | Clostridiales | Lachnospiraceae | Dorea |
| OTU54 | CAG22 | Bacteria | Firmicutes | Clostridia | Clostridiales | Lachnospiraceae | Lachnospiracea_incertae_sedis |
| OTU95 | CAG22 | Bacteria | Firmicutes | Clostridia | Clostridiales | Lachnospiraceae | Clostridium XlVa |
| OTU116 | CAG23 | Bacteria | Firmicutes | Clostridia | Clostridiales | Lachnospiraceae |  |
| OTU492 | CAG23 | Bacteria | Firmicutes | Clostridia | Clostridiales | Lachnospiraceae | Dorea |
| OTU71 | CAG23 | Bacteria | Firmicutes | Clostridia | Clostridiales | Lachnospiraceae |  |
| OTU228 | CAG23 | Bacteria | Firmicutes | Clostridia | Clostridiales | Lachnospiraceae |  |
| OTU336 | CAG23 | Bacteria | Firmicutes | Clostridia | Clostridiales | Lachnospiraceae | Blautia |
| OTU79 | CAG23 | Bacteria | Firmicutes | Clostridia | Clostridiales | Lachnospiraceae | Coprococcus |
| OTU429 | CAG23 | Bacteria | Firmicutes | Clostridia | Clostridiales | Ruminococcaceae | Gemmiger |
| OTU56 | CAG23 | Bacteria | Firmicutes | Clostridia | Clostridiales | Ruminococcaceae | Oscillibacter |
| OTU8 | CAG23 | Bacteria | Firmicutes | Clostridia | Clostridiales | Ruminococcaceae | Gemmiger |
| OTU45 | CAG23 | Bacteria | Firmicutes | Clostridia | Clostridiales | Ruminococcaceae |  |
| OTU70 | CAG23 | Bacteria | Firmicutes | Clostridia | Clostridiales | Ruminococcaceae |  |
